# Structural basis of a nucleosome containing histone H2A.B/H2A.Bbd that transiently associates with reorganized chromatin

**DOI:** 10.1038/srep03510

**Published:** 2013-12-16

**Authors:** Yasuhiro Arimura, Hiroshi Kimura, Takashi Oda, Koichi Sato, Akihisa Osakabe, Hiroaki Tachiwana, Yuko Sato, Yasuha Kinugasa, Tsuyoshi Ikura, Masaaki Sugiyama, Mamoru Sato, Hitoshi Kurumizaka

**Affiliations:** 1 Laboratory of Structural Biology, Graduate School of Advanced Science and Engineering, Waseda University, 2-2 wakamatsu-cho, Shinjuku-ku, Tokyo 162-8480, Japan; 2 Graduate School of Frontier Biosciences, Osaka University, 1-3 Yamadaoka, Suita, Osaka 565-0871, Japan; 3 JST, CREST, 1-3 Yamadaoka, Suita, Osaka, 565-0871, Japan; 4 Graduate School of Medical Life Science, Yokohama City University, 1-7-29 Suehiro-cho, Tsurumi, Yokohama 230-0045, Japan; 5 Department of Mutagenesis, Division of Chromatin Regulatory Network, Radiation Biology Center, Kyoto University, Yoshidakonoe, Sakyo-ku, Kyoto 606-8501, Japan; 6 Research Reactor Institute, Kyoto University, Kumatori, Osaka, 590-0494, Japan; 7 RIKEN SPring-8 Center, 1-1-1 koto, Sayo, Hyogo 679-5148, Japan

## Abstract

Human histone H2A.B (formerly H2A.Bbd), a non-allelic H2A variant, exchanges rapidly as compared to canonical H2A, and preferentially associates with actively transcribed genes. We found that H2A.B transiently accumulated at DNA replication and repair foci in living cells. To explore the biochemical function of H2A.B, we performed nucleosome reconstitution analyses using various lengths of DNA. Two types of H2A.B nucleosomes, octasome and hexasome, were formed with 116, 124, or 130 base pairs (bp) of DNA, and only the octasome was formed with 136 or 146 bp DNA. In contrast, only hexasome formation was observed by canonical H2A with 116 or 124 bp DNA. A small-angle X-ray scattering analysis revealed that the H2A.B octasome is more extended, due to the flexible detachment of the DNA regions at the entry/exit sites from the histone surface. These results suggested that H2A.B rapidly and transiently forms nucleosomes with short DNA segments during chromatin reorganization.

Eukaryotic genomic DNA is organized into chromatin, with the nucleosome as the elemental unit. The protein components of the nucleosome are the core histones H2A, H2B, H3, and H4. In the canonical nucleosome, two each of the H2A–H2B and H3–H4 dimers associate as the histone octamer, and 145–147 base pairs of DNA are wrapped around it^1^,^2^,^3^. In general, nucleosomes are biochemically very stable structures *in vitro*, but they are dynamically reorganized during transcription, replication, recombination, and repair in living cells^4^,^5^,^6^,^7^. During the nucleosome remodeling and assembly/disassembly processes, unusual nucleosomes, such as a hexasome lacking one H2A–H2B dimer from the canonical nucleosome (octasome), are also formed, especially during the transcription initiation and elongation steps^8^,^9^,^10^,^11^,^12^. This structural plasticity of the nucleosome appears to be essential for gene expression and genomic DNA maintenance.

Recent studies have revealed that non-allelic variants of histones exhibit distinct dynamic behaviors. The structural basis underlying the differential nucleosome stabilities of H3 variants has been extensively studied^13^,^14^,^15^,^16^,^17^. Among nucleosomes containing human H3 variants, the H3T (H3.4) nucleosome is extremely labile both *in vitro* and *in vivo*^14^, and the centromere-specific H3 variant, CENP-A (cenH3), forms an unusual nucleosome structure, in which the DNA segments located at the entry/exit sites are flexibly detached from the histone surface^16^. Therefore, the natural amino acid substitutions in histone variants contribute toward the diversity of nucleosome structures and dynamics, in combination with post-translational modifications^18^,^19^,^20^.

In addition to the H3 variants, several H2A variants have been identified in humans, including H2A.X, H2A.Z, macroH2A, and H2A.B (H2A.Bbd; Barr-body deficient)^21^,^22^. H2A.B is a distant H2A variant, and shares about 50% amino acid identity with the canonical H2A^23^,^24^. In living cells, H2A.B tagged with the green fluorescent protein (GFP) is highly mobile, as compared to the canonical H2A tagged with GFP^25^. Chromatin immunoprecipitation coupled with genomic microarray or next generation sequencing analyses revealed that H2A.B is preferentially localized on the bodies of actively transcribed genes^26^,^27^. Biochemical studies demonstrated that H2A.B is efficiently incorporated into nucleosomes, and the DNA segments at the entry/exit sites of the H2A.B nucleosome are more susceptible to endonuclease^28^,^29^. Consistently, electron microscopic and atomic force microscopic analyses indicated that the DNA entry/exit sites are more flexible in the H2A.B nucleosome, as compared to the canonical H2A nucleosome^29^,^30^. The H2A.B nucleosome has a propensity to form open chromatin in the nucleosome array^31^. Based on these studies, H2A.B has been implicated in the nucleosome dynamics associated with chromatin opening.

In the present study, we further analyzed the properties of H2A.B in *de novo* assembly. In living cells, GFP-H2A.B transiently accumulated at DNA replication and repair foci. In a reconstituted system, we found that H2A.B forms two types of nucleosomes (i.e., octasome and hexasome) with shorter DNA fragments, such as a 124 bp DNA, which allows only hexasome formation with the canonical H2A. A small-angle X-ray scattering analysis revealed that the H2A.B octasome structure contained extra volumes on both sides of the nucleosome, as compared to the canonical H2A octasome, probably reflecting the flexible DNA segments at the entry/exit sites. These data suggested that H2A.B may form an intermediate nucleosome with a shorter DNA segment, when chromatin is newly formed or reorganized after DNA replication, repair, and transcription.

## Results

### Behavior of GFP-H2A.B during the cell cycle

**Figure 1:**
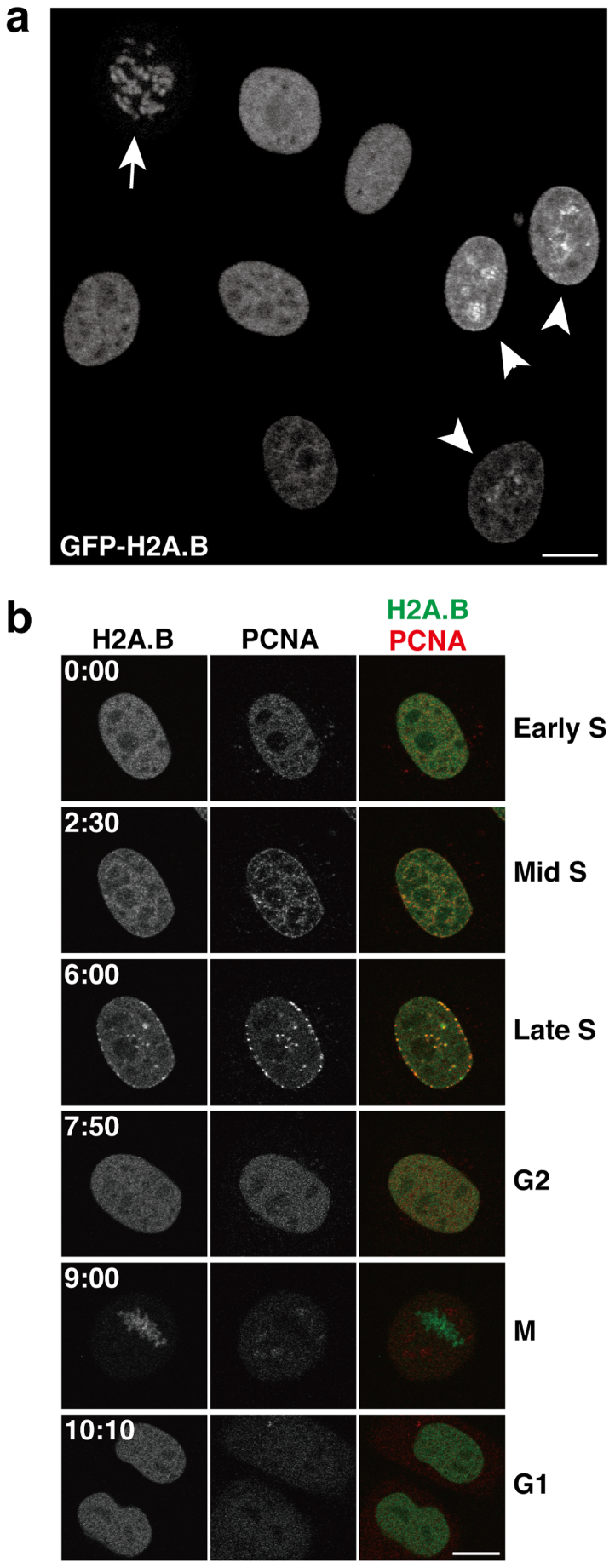
Localization of GFP-H2A.B in living cells. (a) GFP-H2A.B distribution in living HeLa cells imaged by confocal microscopy. GFP-H2A.B is localized to interphase nuclei and mitotic chromosomes (arrow). In some nuclei, GFP-H2A.B is concentrated in foci (arrowheads). (b) GFP-H2A.B is concentrated in replication foci during S phase. HeLa cells expressing both GFP-H2A.B and PCNA-mCherry were grown on a glass-bottom dish under a confocal microscope. A cell exhibiting the early replication pattern of PCNA-mCherry was chosen for time-lapse imaging. Confocal images of GFP-H2A.B and PCNA-mCherry were obtained every 5 min (see Supplementary Movie S1). The elapsed time (hh:mm) from the start of recording is shown on each panel. During mid- to late-S phase, GFP-H2A.B becomes clearly concentrated in PCNA foci, where DNA replication occurs. Bars, 10 μm.

We first established HeLa cells stably expressing GFP-H2A.B. GFP-H2A.B was localized to the chromosomes in mitotic cells (Fig. 1a, arrow) and distributed in euchromatin in interphase nuclei (Fig. 1a), as previously shown^23^ and suggested by chromatin immunoprecipitation analyses^26^,^27^. Within a cell population, however, cells exhibiting GFP-H2A.B concentrated in discrete foci were also observed (Fig. 1a, arrowheads). Since these foci resembled DNA replication foci^32^, we investigated the localization of GFP-H2A.B during the cell cycle. We established cells expressing both GFP-H2A.B and PCNA-mCherry, as a marker of DNA replication complexes formed during S phase^32^. Time-lapse microscopy revealed that GFP-H2A.B became more concentrated in PCNA foci during S phase (Fig. 1b; Supplementary Movie S1). These results suggested that H2A.B is assembled into chromatin just after DNA replication, and is soon replaced with the canonical H2A. This is consistent with the lower stability of nucleosomal H2A.B in living cells, as previously detected by fluorescence recovery after photobleaching (FRAP)^25^.

**Figure 2:**
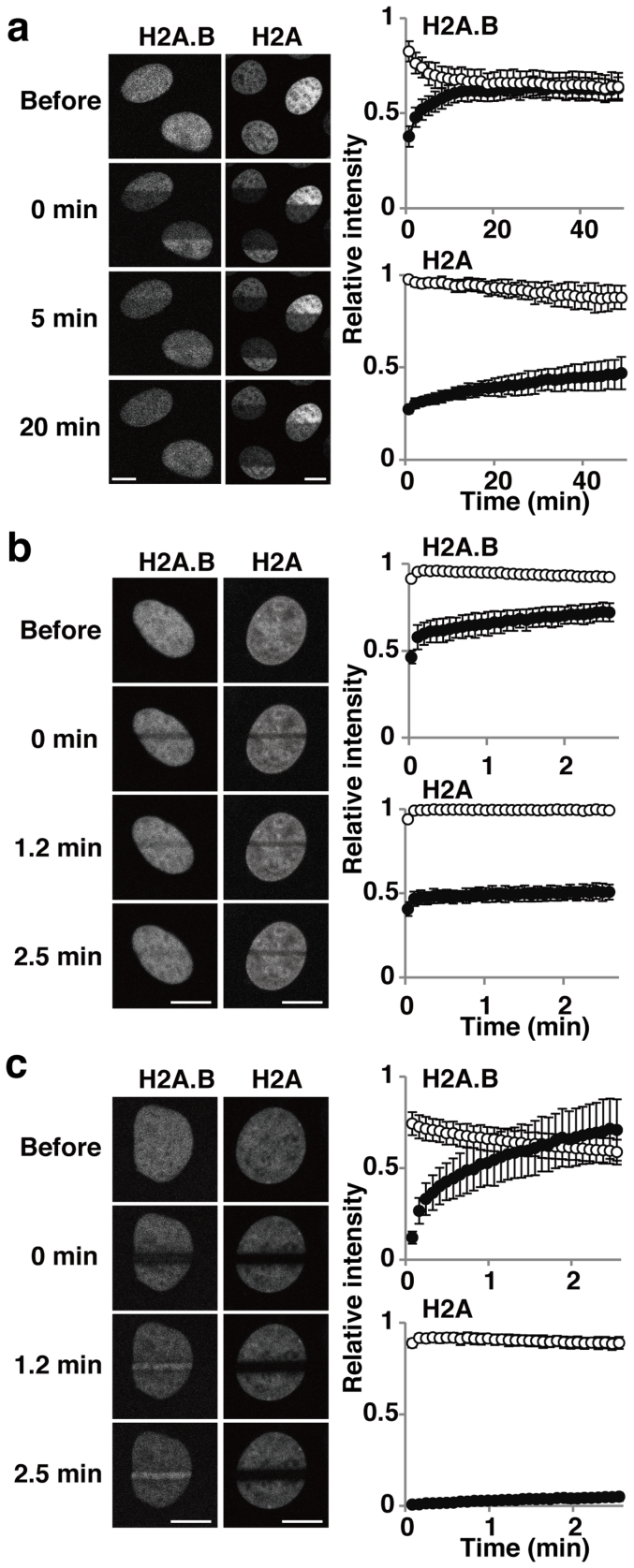
Kinetics of GFP-H2A.B in living cells. The kinetics of GFP-H2A.B and GFP-H2A were analyzed by bleaching with a 488-nm laser (a and b), and by irradiation with a 405-nm laser to induce DNA damage (c). Fluorescence intensities of bleached or damaged (closed circles) and unbleached or undamaged (open circles) areas are plotted as relative values to the initial intensity of the same area before bleaching or damaging (averages with standard deviations; N = 14–24). (a) One-half of the nucleus was bleached using a 488-nm laser for ~38 s, and fluorescence images were obtained for 50 min. The first post-bleach image was acquired at ~45 s after the beginning of bleaching. (b) A 2 μm strip was bleached using a 488-nm laser for 110 ms, and fluorescence images were obtained for 2.5 min. The first post-bleach image was acquired at 1.8 s after the beginning of bleaching. (c) A 2 μm strip was irradiated using a 405-nm laser for 3.6 s, and fluorescence images were obtained for 2.5 min. The first post-bleach image was acquired at 4.9 s after the beginning of irradiation. Bars, 10 μm.

We also confirmed the rapid recovery of GFP-H2A.B, as compared to the canonical GFP-H2A, after photobleaching with a 488-nm laser line (Fig. 2a and b). After bleaching one-half of the nucleus, the exchange of GFP-H2A.B was almost complete (i.e., the intensities of the bleached and unbleached areas reached the same level) within 20 min, while only subtle recovery in the bleached area was observed with GFP-H2A (Fig. 2a). Although the expression levels of these GFP-tagged histones varied from cell to cell, their FRAP recovery kinetics did not depend on the original fluorescence intensity (Supplementary Fig. S1), probably because the GFP-tagged versions are equilibrated with the endogenous histone molecules that are present in great excess^4^. The FRAP data also revealed the difference in the amounts of chromatin-free, diffusible fractions between GFP-H2A.B and GFP-H2A. If free molecules are present, they diffuse in and out of the bleached area during bleaching, and this causes the intensity of the unbleached area to decrease from the original level, in a phenomenon called fluorescence loss in photobleaching (4; and references therein). In addition, under the slow acquisition conditions used, free unbleached molecules can diffuse into the bleached area before the acquisition of the first post-bleach image. Under long (~39 s) bleaching conditions (Fig. 2a), the post-bleach intensity of GFP-H2A.B in the unbleached area dropped to 0.83, while that of GFP-H2A was close to the original level (0.98). Consistently, the post-bleach intensity of GFP-H2A.B in the bleached area was higher than that of GFP-H2A (0.38 vs. 0.27; Fig. 2a). These data suggested the presence of a large pool of chromatin-free GFP-H2A.B, in contrast to GFP-H2A. The rapid recovery and the larger free pool of GFP-H2A.B were also observed in short-term FRAP experiments (Fig. 2b). Under these rapid (110 ms) strip-bleach conditions, the post-bleach intensity in the unbleached region was not affected, but the intensity of GFP-H2A.B in the bleached area was higher than that of GFP-H2A.

As GFP-H2A.B is concentrated in replication foci during S phase, the FRAP recovery kinetics could depend on the cell cycle. To clearly distinguish S-phase cells from non-S phase (G1 or G2) cells, we used HeLa cells expressing both GFP-H2A.B and PCNA-mCherry, a definitive indicator of replication foci^32^. GFP-H2A.B in non-S phase cells exhibited higher recovery than in S phase cells, but this difference was due to the different amounts in the free pools (i.e., the level at the first post-bleach point), and the curve shapes (exchange rates) were very similar (Supplementary Fig. S2). The lower level of the chromatin-free fraction in S phase is likely to be a consequence of the massive assembly of GFP-H2A.B into replicated chromatin in addition to the basal level of incorporation into transcribed chromatin^26^,^27^. As the FRAP curve shapes were similar between S and non-S phase cells, the dissociation rates are apparently comparable even in replication-coupled assembly.

Interestingly, when cells were microirradiated using a 405-nm laser line that can induce DNA damage, GFP-H2A.B accumulated in the irradiated area within 2 min (Fig. 2c). In contrast, the canonical GFP-H2A remained relatively immobile under the same conditions (Fig. 2c), similar to the results from photobleaching using a 488-nm laser line (Fig. 2b). Once again, the curve shapes were similar between S and non-S phase cells (Supplementary Fig. S3). These results suggested that H2A.B is specifically assembled into chromatin during DNA damage repair. This may simply be due to the presence of more chromatin-free H2A.B molecules than H2A; however, specific mechanisms might also be involved. Although the deposition complex of H2A.B has not been characterized yet, a specific chaperone may facilitate the assembly of H2A.B to the damaged chromatin. Alternatively, H2A.B incorporation might be coupled with H2AX evicion, which occurs after DNA damage in a ubiquitylation-dependent manner^33^.

Taken together with previous data^26^,^27^, H2A.B appears to be transiently assembled into chromatin after DNA replication, DNA repair, and transcription, which all involve chromatin reorganization, including H2A-H2B dimer exchange^4^,^5^,^8^,^12^. To obtain mechanistic insights into the transient H2A.B assembly, we performed structural and biochemical analyses using a reconstituted system.

### *De novo* nucleosome formation by H2A.B with 116, 124, and 130 bp DNAs

**Figure 3:**
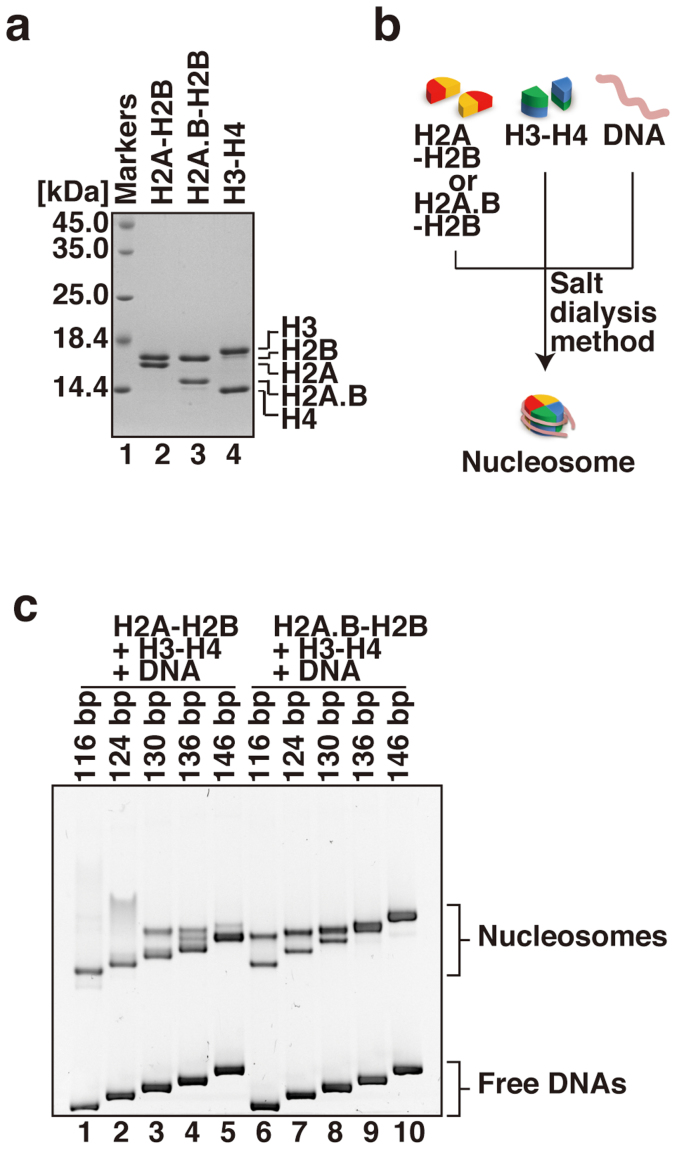
Reconstitution of the H2A.B and H2A nucleosomes with various lengths of DNAs. (a) Purified human recombinant histone complexes were analyzed by 18% SDS-PAGE with Coomassie Brilliant Blue staining. (b) Schematic representation of the nucleosome reconstitution experiments by the salt dialysis method. (c) Nucleosomes reconstituted with 116 bp (lanes 1 and 6), 124 bp (lanes 2 and 7), 130 bp (lanes 3 and 8), 136 bp (lanes 4 and 9), and 146 bp (lanes 5 and 10) DNAs were analyzed by non-denaturing 6% PAGE, and the gel was stained with ethidium bromide after PAGE. Lanes 1–5 indicate the nucleosomes reconstituted with H2A–H2B and H3–H4, and lanes 6–10 indicate the nucleosomes reconstituted with H2A.B–H2B and H3–H4. The 146 bp DNA was the palindromic satellite DNA derivative.

Previous reports found that, in the H2A.B nucleosome, about 118–130 bp of DNA are protected from digestion by micrococcal nuclease (MNase)^28^,^29^. This abbreviated contact of DNA with histones may explain the instability of H2A.B once it is assembled within a nucleosome; however, it remained uncertain whether H2A.B could form a nucleosome with a short DNA segment *de novo*. To determine whether the H2A.B nucleosome is actually assembled with short DNA fragments, we performed nucleosome reconstitutions with various lengths of DNA fragments (116, 124, 130, 136, and 146 bp DNAs) and human histones purified as bacterially expressed recombinant proteins (Fig. 3a). The nucleosome reconstitution was performed with H2A.B–H2B (or H2A–H2B) and H3–H4 by the salt-dialysis method (Fig. 3b). As expected, both H2A and H2A.B formed a nucleosome with 146 bp DNA (Fig. 3c, lanes 5 and 10). However, we found that H2A.B formed two types of nucleosomes, which migrated differently on native PAGE when a 116, 124 or 130 bp DNA fragment was used as the substrate for nucleosome reconstitution (Fig. 3c, lanes 6–8). In contrast, H2A only formed one type of nucleosome when a 116 or 124 bp DNA fragment was used (Fig. 3c, lanes 1 and 2). When the nucleosome reconstitution was performed with H2A in the presence of a 130 or 136 bp DNA fragment, extra bands, with slower migration than the major (lower) band, were detected (Fig. 3c, lanes 3 and 4). These extra bands may correspond to the tetrasome containing H3–H4 and/or the complex formed by improper histone-DNA binding (Supplementary Fig. S4).

### H2A.B forms both an octasome and a hexasome *de novo* with a 124 bp DNA fragment

**Figure 4:**
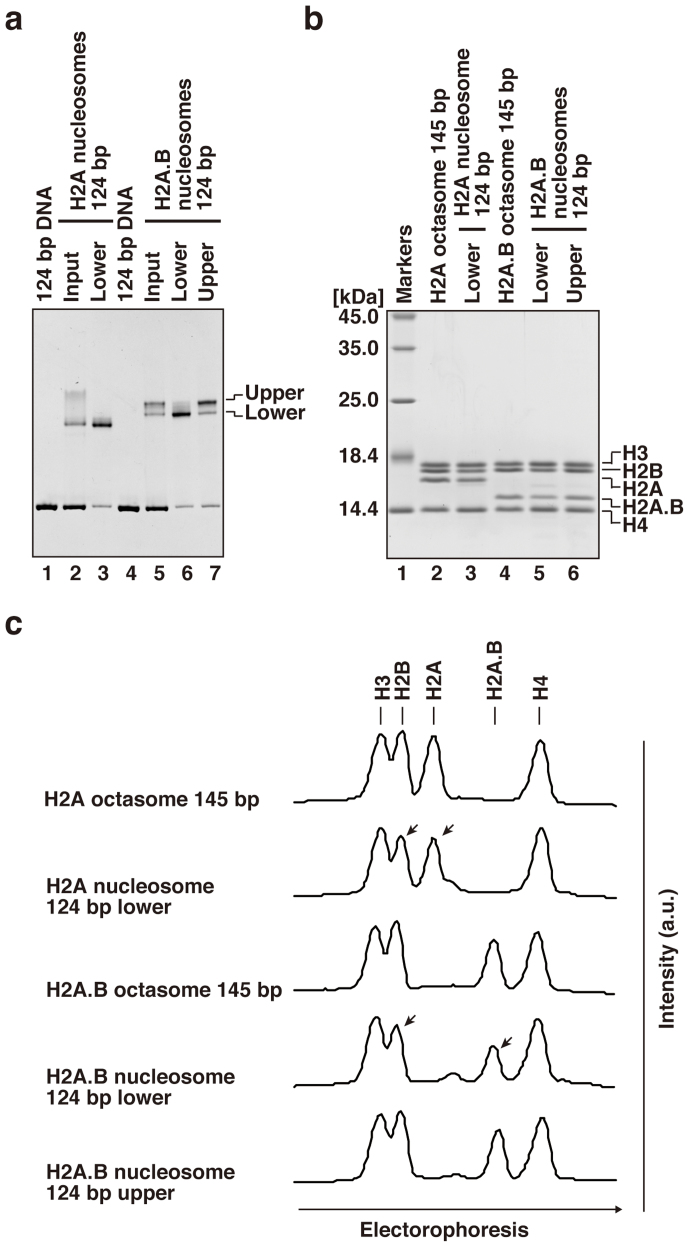
H2A.B forms both an octasome and a hexasome with a 124 bp DNA fragment. (a) Nucleosomes reconstituted with a 124 bp DNA were purified using a Prep Cell apparatus, and were analyzed by non-denaturing 6% PAGE, and the gel was stained with ethidium bromide after PAGE. Lanes 1 and 4 indicate the 124 bp DNA used in the nucleosome reconstitution. Lanes 2 and 3 indicate the H2A nucleosome samples before (input) and after (lower band) Prep Cell purification, respectively. Lane 5 indicates the H2A.B nucleosome sample before (input) purification, and lanes 6 (lower band) and 7 (upper band) indicate the H2A.B nucleosomes after Prep Cell purification. (b) The histone compositions of the purified nucleosomes were analyzed by 18% SDS-PAGE with Coomassie Brilliant Blue staining. Lane 1 indicates molecular mass markers. Lanes 2 and 4 represent the histones included in the purified fractions of the H2A and H2A.B octasomes reconstituted with a 145 bp 601 DNA fragment, respectively. Lane 3 depicts the histones included in the purified fraction of the H2A nucleosome with a 124 bp DNA fragment, as shown in panel a, lane 3. Lanes 5 and 6 show the histones included in the purified fractions of the H2A.B nucleosomes with a 124 bp DNA fragment. Lanes 5 and 6 indicate the lower and upper band fractions, as shown in panel a, lanes 6 and 7, respectively. (c) Line intensity profiles (a.u.) of the gel shown in (b). Arrowheads indicate bands with decreased intensity, due to the lack of a single H2A–H2B or H2A.B–H2B dimer in the hexasome.

To determine the histone compositions of the H2A and H2A.B nucleosomes reconstituted with a 124 bp DNA fragment, we electrophoretically purified the upper and lower bands of the reconstituted H2A.B nucleosome (Fig. 4a). The histone compositions were analyzed by SDS-PAGE and Coomassie Brilliant Blue staining (Fig. 4b). Consistent with our previous study^34^, the H2A nucleosome reconstituted with a 124 bp DNA fragment contained about one-half the amount of H2A–H2B, as compared to that of H3–H4 (Fig. 4b, lane 3, and c), indicating hexasome formation. Similarly, the lower band of the H2A.B nucleosome with a 124 bp DNA fragment contained a reduced amount (about 50%) of H2A.B–H2B (Fig. 4b, lane 5, and c). In contrast, the upper band contained similar amounts of H2A.B–H2B (Fig. 4b, lane 6, and c) relative to the control H2A.B octasome (Fig. 4b, lane 4, and c). These results indicated that H2A.B forms both hexasomes and octasomes *de novo*, but H2A only forms a hexasome with a shorter DNA fragment, such as 124 bp.

### Solution structure of the H2A.B nucleosome

**Figure 5:**
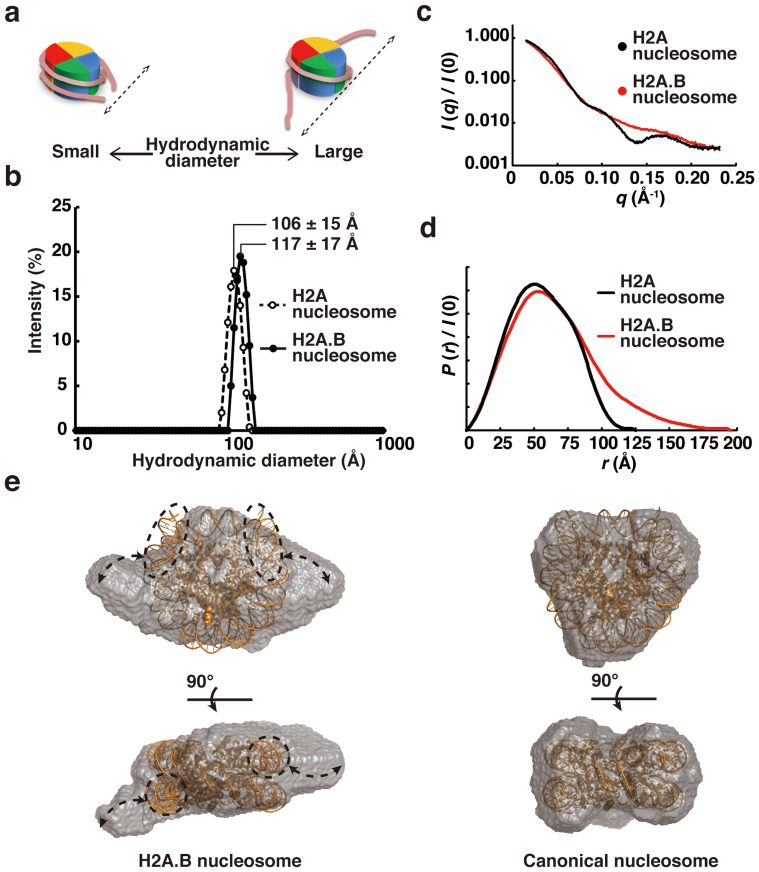
Solution structure of the H2A.B octasome. (a) Schematic representations of the relationship between the hydrodynamic diameter in the DLS analysis and the nucleosome structure. (b) Particle size distribution profiles in the DLS analysis of the H2A and H2A.B octasomes with a 145 bp 601 DNA fragment. Closed and open circles represent the particle size distributions of the H2A.B and H2A octasomes, respectively. The nucleosome concentration was 1.0 mg DNA/ml. The hydrodynamic diameters (Z-average size) of the H2A and H2A.B octasomes were 10.598 nm (error: 1.4658 nm) and 11.682 nm (error: 1.6588 nm), respectively. Both octasomes were detected as monodisperse. (c) The SAXS intensity curves of H2A and H2A.B octasomes with a 145 bp DNA fragment. (d) Distance distribution functions *P*(*r*) of the H2A and H2A.B octasomes. (e) Dummy atom models of the H2A.B octasome (left) and the H2A octasome (right). Side and top views are presented in the upper and lower panels, respectively. The crystal structure of the canonical nucleosome (PDB ID: 3AFA) is superimposed on the solution structures of the H2A and H2A.B octasomes. Putative flexible DNA segments are enclosed by dashed circles. Arrows indicate the extra volumes, which may be the mobile areas of the flexible DNA segments.

To gain further insights into the structural differences between the H2A.B nucleosome and the canonical H2A nucleosome, we performed dynamic light scattering (DLS) measurements. In this analysis, we used the 145 bp 601 DNA fragment, which reportedly forms a stably positioned nucleosome^3^. Consistent with previous electron and atomic force microscopic analyses^29^, the mean particle size of the H2A.B octasome with the 145 bp 601 DNA fragment was clearly larger than that of the canonical H2A octasome (Fig. 5a and b). These DLS data suggested that the DNA segments may be unwrapped at the entry/exit sites of the H2A.B octasome. We then performed a small-angle X-ray scattering (SAXS) analysis of the H2A.B octasome with the 145 bp 601 DNA fragment (Fig. 5c, d, and e). The radius of gyration (*R*_g_ = 49.4 ± 0.4 Å) and the maximum diameter (*D*_max_ = 195 Å) of the H2A.B octasome were significantly larger than those of the canonical H2A octasome (*R*_g_ = 43.4 ± 0.3 Å, *D*_max_ = 125 Å). The shape of the distance distribution function (*P*(*r*)) of the H2A.B octasome has a long tail, suggesting that the structure of the H2A.B octasome is not compact. Based on these data, we constructed a dummy atom model of the H2A.B octasome structure (Fig. 5e). In this model, extra volumes were observed on both sides of the H2A.B octasome, as compared to the canonical H2A octasome model. These extra volumes may reflect the flexible DNA segments at the entry/exit sites of the H2A.B octasome. It should be noted that nucleic acids scatter more strongly than proteins. Despite the differences in X-ray scattering by nucleic acids and proteins, the known crystal structure of the canonical nucleosome and the SAXS envelope are consistent with each other (Fig. 5e, right panels)^35^. This indicated that the SAXS data could be used to build low-resolution structural models of nucleosomes.

## Discussion

H2A.B (originally named H2A.Bbd) was found as a distant H2A variant that is excluded from the inactive X-chromosome (Barr body)^23^. A genome-wide analysis revealed that H2A.B is preferentially associated with actively transcribed genes^26^,^27^. It was previously reported that 118–130 bp DNAs are protected in the H2A.B nucleosome from MNase digestion^28^,^29^. In the present study, we found that H2A.B intrinsically forms both a hexasome and an octasome with shorter DNA segments, such as 116–130 bp. In contrast, the canonical H2A did not form an octasome with a 124 bp DNA fragment. Therefore, the octasome formation with shorter DNA segments may be a specific characteristic of the H2A.B nucleosome. This property of H2A.B might facilitate rapid and transient nucleosome reformation with shorter DNA segments available after transcription, replication, and repair, before more stable nucleosomes are formed with the canonical H2A. The transient formation of H2A.B nucleosomes may protect DNA, by preventing the access of specific or non-specific DNA binding proteins that can alter the chromatin structure and/or epigenetic status. The alteration of the linker DNA orientation, which is caused by DNA flexibility at the entry/exit regions of the nucleosome, may also affect linker histone binding. Indeed, the H2A.B nucleosome is reportedly defective in linker histone H1 binding^30^. Histone H1 binds to nucleosomal and linker DNAs, and forms a stem-like structure^36^ to establish a higher-order chromatin structure. H2A.B nucleosomes may prevent histone H1 binding and maintain the open chromatin structure after temporary nucleosome disruption by transcription, replication, and repair, although H2A.B may play more specific roles^27^.

H2A.B is reportedly expressed in spermatogenic cells and the nucleosomal chromatin fraction of human sperm^37^. H2A.B does not form a stable histone octamer without DNA^28^, and nucleosomal H2A.B exchanges rapidly in nuclei^25^. Consistently, H2A.B confers a more flexible nucleosome structure, as compared to the other mammalian histone H2A variants, H2A.X, H2A.Z, macroH2A, and canonical H2A^38^. Intriguingly, these characteristics are common to human histone H3T^14^, which is also highly expressed in testis^39^,^40^,^41^. Therefore, the unstable nature of the testis-specific nucleosomes may play an essential role in the formation of the specific chromatin structure packaged within the sperm nucleus.

## Methods

### Cells and microscopy

HeLa cells were routinely grown in Dulbecco's modified Eagle's medium (DMEM; Nacalai Tesque) containing antibiotics (10 U/ml penicillin and 50 μg/ml streptomycin; Sigma-Aldrich) and 10% fetal calf serum (FCS). Cells expressing GFP-H2A.B were selected in 1 mg/ml G418 (Nacalai Tesque) after transfection with the GFP-H2A.B expression vector, which was constructed by inserting a PCR product into the pEGFP-C1 vector (Clontech). The cells expressing GFP-H2A were previously described^42^. Cells expressing both PCNA-mCherry and GFP-H2A.B or GFP-H2A were generated by cell fusion^32^,^43^. HeLa cells expressing GFP-H2A.B or GFP-H2A (G418 resistant) and those expressing PCNA-mCherry (puromycin resistant) were fused using polyethylene glycol (Roche), and single colonies were selected in the presence of 1 mg/ml G418 and 0.5 μg/ml puromycin.

For live cell analysis, cells were grown on a glass-bottom dish (Mat-Tek) without synchronization. When necessary, the PCNA-mCherry pattern was used to judge the cell cycle point of each cell. The dish was placed on a confocal microscope (FV-1000; Olympus), equipped with a culture system (Tokai Hit) at 37°C under a 5% CO_2_ atmosphere. For time-lapse imaging, confocal images were collected every 5 min for 14 h, using a PlanApoN 60× OSC (NA = 1.4) oil-immersion lens (512 × 512 pixels; 4 μs/pixel; 4 line Kalman; pinhole 110 μm; line sequential scanning with 488- and 543-nm lasers). Fluorescence recovery after photobleaching (FRAP) and laser micro-irradiation were performed using a confocal microscope (FV-1000; Olympus) with a PlanApoN 60× OSC (NA = 1.4) oil-immersion lens. For Fig. 2a, after 2 images were obtained every 30 s (0.3% 488-nm laser transmission; 4 μs/pixel; 512 × 512 pixels; pinhole 800 μm; 1.5× zoom), one-half of each nucleus was bleached (100% 488-nm laser transmission; 20 μs/pixel; 2 iterations), and images were obtained using the original settings. For Fig. 2b and 2c, after 5 images were obtained (0.1% 488-nm laser transmission; 4 μs/pixel; 512 × 512 pixels; pinhole 800 μm; 8× zoom), a 2 μm width strip was bleached (100% 488-nm laser transmission with 4 μs/pixel for FRAP, or 100% 405-nm laser transmission with 200 μs/pixel for micro-irradiation), and 95 more images were obtained using the original settings. Fluorescence intensity measurements were performed using Image J version 1.45 s (http://rsb.info.nih.gov/ij/). The net intensities of the bleached and unbleached areas were obtained by subtracting the background intensity outside nuclei in each time frame. To obtain relative intensities to the initial intensity of the same area, the net intensities were normalized to the average intensity of pre-bleach images.

### Purification of recombinant human H2A, H2B, H3.1, and H4

Human histones H2A, H2B, H3.1 and H4 were expressed as the N-terminally His_6_-tagged proteins in *Escherichia coli* cells^44^, and purified according to the method described previously^15^,^16^,^14^. Briefly, the His_6_-tagged histones were recovered from the insoluble fraction in 50 mM Tris-HCl buffer (pH 8.0), containing 7 M guanidine hydrochloride, 500 mM NaCl, and 5% glycerol, and purified by Ni-NTA agarose chromatography (Qiagen) under denaturing conditions. The His_6_-tag was then removed by cleavage with thrombin protease (1 unit/mg of histones; GE Healthcare), and further purified by Mono S column chromatography (GE Healthcare). The purified histones were dialyzed against water, freeze-dried, and stored at 4°C.

### Preparation of the H2A.B–H2B complex

The human H2A.B gene was amplified from a human testis cDNA pool (Clontech) by polymerase chain reaction (PCR). The resulting DNA fragment was ligated into the same vector used for the H2A expression^44^. The recombinant H2A.B was expressed in *Escherichia coli* BL21(DE3) codon(+)RIL cells (Stratagene), as the N-terminally His_6_-tagged protein. The His_6_-tagged H2A.B was recovered from the insoluble fraction in 50 mM Tris-HCl buffer (pH 8.0), containing 7 M guanidine hydrochloride, 500 mM NaCl, and 5% glycerol, and purified by Ni-NTA agarose chromatography (Qiagen) under denaturing conditions. The purified His_6_-tagged H2A.B was mixed with purified H2B (1 mg protein/ml) at a 1:1 stoichiometry, and the sample was dialyzed against 20 mM Tris-HCl buffer (pH 7.5), containing 7 M guanidine-HCl and 20 mM 2-mercaptoethanol, for 4 hours, followed by overnight dialysis against 10 mM Tris-HCl buffer (pH 7.5), containing 2 M NaCl, 1 mM EDTA, and 2 mM 2-mercaptoethanol. The sample was then dialyzed against the buffers containing 1 M NaCl for 4 hours, 0.5 M NaCl for 4 hours, and 0.1 M NaCl overnight. The His_6_-tag was removed from H2A.B by cleavage with thrombin protease (1 unit/mg of His_6_-tagged H2A.B). The H2A.B–H2B complex was further purified by Superdex200 (GE Healthcare Biosciences) gel filtration chromatography with 10 mM Tris-HCl buffer (pH 7.5), containing 2 M NaCl, 1 mM EDTA and 2 mM 2-mercaptoethanol*.*

### Preparation of the H2A–H2B and H3.1–H4 complexes

The H2A–H2B and H3.1–H4 complexes were reconstituted according to the previously published methods^14^,^15^,^16^. Purified H2A and H2B were mixed (1 mg protein/ml) at a 1:1 stoichiometry in 20 mM Tris-HCl buffer (pH 7.5), containing 7 M guanidine-HCl and 20 mM 2-mercaptoethanol. Purified H3.1 and H4 were also mixed (1 mg protein/ml) at a 1:1 stoichiometry in 20 mM Tris-HCl buffer (pH 7.5), containing 7 M guanidine-HCl and 20 mM 2-mercaptoethanol. These H2A–H2B and H3.1–H4 mixtures were dialyzed against 10 mM Tris-HCl buffer (pH 7.5), containing 2 M NaCl, 1 mM EDTA, and 2 mM 2-mercaptoethanol. The resulting histone H2A–H2B and H3.1–H4 complexes were further purified by Superdex200 (GE Healthcare Biosciences) gel filtration chromatography.

### Preparation of 116–146 bp DNA fragments for nucleosome reconstitution

The 116 bp, 124 bp, 130 bp, 136 bp, and 146 bp DNA fragments, containing the α-satellite sequence, were prepared by self-ligation of the 56 bp, 60 bp, 63 bp, 66 bp, and 71 bp DNA segments containing an additional four-base overhang at one 5′ end (proximal end), which is located at the center of the DNA fragment after self-ligation. The 56 bp, 60 bp, 63 bp, and 66 bp DNAs were produced by deleting 15 bp, 11 bp, 8 bp, and 5 bp from the distal end of the 71 bp DNA, respectively. These DNA fragments were bacterially produced, and were purified by the method described previously^45^. Sixteen repeats of each of these DNA segments were inserted into the pGEM-T Easy Vector (Promega). The plasmids were amplified in *E. coli* cells. The DNA fragments containing the α-satellite sequence were isolated from the plasmid vector by digestion with *Eco*RV. The ends of the *Eco*RV fragments were dephosphorylated with calf intestine alkaline phosphatase (CIAP; Nippon Gene) treatment. After the CIAP treatment, the proximal end was digested with *Eco*RI, and the four-base overhang was created. Each of these DNA fragments was self-ligated, and the 116 bp, 124 bp, 130 bp, 136 bp, and 146 bp DNA fragments were prepared.

The 145 bp 601 DNA was bacterially produced, and was purified by the method described previously^45^,^46^. Eight 145 bp 601 DNA fragments were tandemly ligated into the pGEM-T Easy vector. The purified plasmid containing eight 601 DNA repeats was digested with *Eco*RV, and the resulting 145 bp 601 DNA fragment was treated with CIAP.

### Reconstitution of nucleosomes

The nucleosomes were reconstituted by the salt dialysis method, as previously described^14^. H2A–H2B or H2A.B–H2B and H3–H4 were mixed with a DNA fragment (5.2 μM of the molecule for biochemical experiments or 6.8 μM of the molecule for DLS and SAXS analyses), at a molar ratio of H2A–H2B (or H2A.B–H2B):H3–H4:DNA = 2:2.6:1, in a solution containing 2 M KCl. The KCl concentration was gradually reduced to 250 mM with a peristaltic pump. The reconstituted nucleosomes were incubated at 55°C for 2 hours, to eliminate the non-specific histone-DNA binding. The nucleosomes were further purified by non-denaturing polyacrylamide gel electrophoresis (PAGE), using a Prep Cell apparatus (Bio-Rad). The nucleosomes were eluted with 20 mM Tris-HCl buffer (pH 7.5), containing 1 mM EDTA and 1 mM dithiothreitol.

### Dynamic light scattering (DLS) measurements

DLS measurements of the nucleosomes (1.0 mg DNA/ml) containing H2A.B or H2A were performed at 20°C with a Zetasizer Nano μV system (Malvern Instruments), in 20 mM Tris-HCl buffer (pH 7.5), containing 1 mM EDTA and 1 mM dithiothreitol. For the DLS experiments, the 145 bp 601 DNA was used.

### Small-angle X-ray scattering (SAXS) analysis

The SAXS camera (SAXES) installed at BL10C of KEK-PF (Ibaraki, Japan) was used to collect SAXS intensity data. The SAXS intensities of H2A and H2A.B nucleosome solutions were measured with an R-AXIS IV^++^ imaging plate detector at 20°C with an exposure time of 600 s and a sample-to-detector distance of 2040.060 mm, which was calibrated by the powder diffraction from silver docosanoate. The nucleosome samples were reconstituted with the 145 bp 601 DNA. Circular averaging of the SAXS intensity data was then performed to obtain the one-dimensional intensity data *I*(*q*) as a function of *q* (*q* = 4πsin*θ*/λ, where 2*θ* is the scattering angle and the X-ray wavelength λ = 1.488 Å). To correct the inter-particle interference effect, *I*(*q*) data were collected at three nucleosome concentrations (0.5, 1.0, and 3.0 mg/mL), and were extrapolated to zero concentration. A SAXS intensity measurement of the buffer solution (20 mM Tris-HCl buffer, pH 7.5, containing 1 mM EDTA and 1 mM dithiothreitol) for background subtraction was also performed, using the same conditions and procedure as those used for the nucleosome solutions.

The radius of gyration, *R*_g_, was estimated by fitting the *I*(*q*) data using the Guinier approximation *I*(*q*) = *I*(0) exp(−*q*^2^*R*_g_^2^/3), where *I*(0) is the forward scattering at the zero scattering angle, in the smaller angle region of *qR*_g_ < 1.3. The error of *R*_g_ was estimated from the least-squares fitting. The distance distribution function *P*(*r*) and its error were calculated by the program GNOM^47^. The maximum dimension, *D*_max_, was estimated from the *P*(*r*) function as the distance *r*, where *P*(*r*) = 0, and its error was estimated from the errors of the *P*(*r*) values around *P*(*r*) = 0. All data were processed and analyzed using the software applications embedded in the ATSAS package^48^.

### Construction of dummy atom models of nucleosomes containing H2A.B or H2A

The low-resolution dummy atom models of the nucleosomes containing H2A.B or H2A were produced by DAMMIN, using the scattering data^49^. Ten independently calculated, low-resolution dummy atom models were averaged with DAMAVER for each nucleosome^50^.

## Author Contributions

Y.A., K.S., A.O. and H.T. purified the histones, and performed biochemical analyses. H. Kimura, Y.S. and Y.K. performed the cell-based analysis. Y.A., T.O., M. Sugiyama and M. Sato performed the SAXS analysis. T.I. contributed to the preliminary FRAP analysis. H. Kimura and H. Kurumizaka conceived the study, supervised all of the work, and wrote the manuscript.

## Supplementary Material

Supplementary Information Supplementary movie

Supplementary Information Supplementary Figures
