# Supplementary material for: Structural basis of a nucleosome containing histone H2A.B/H2A.Bbd that transiently associates with reorganized chromatin
Source: Sci Rep. 2013 Dec 16;3:3510. doi: 10.1038/srep03510 (PMC3863819; doi:10.1038/srep03510)
Supplement: Supplementary Information — Supplementary Figures [file srep03510-s2.pdf]

Supplementary Figures

Structural basis of a nucleosome containing histone H2A.B/H2A.Bbd that transiently associates with reorganized chromatin

Yasuhiro Arimura[1], Hiroshi Kimura[2,3,*], Takashi Oda[4], Koichi Sato[1], Akihisa Osakabe[1], Hiroaki Tachiwana[1], Yuko Sato[2,3], Yasuha Kinugasa[2], Tsuyoshi Ikura[5], Masaaki Sugiyama[6], Mamoru Sato[4,7], and Hitoshi Kurumizaka[1,*]

[1] Laboratory of Structural Biology, Graduate School of Advanced Science and Engineering, Waseda University, Shinjuku-ku, Tokyo 162-8480, Japan.

[2] Graduate School of Frontier Biosciences, Osaka University, 1-3 Yamadaoka, Suita, Osaka 565-0871, Japan.

[3] JST, CREST, 1-3 Yamadaoka, Suita, Osaka, 565-0871, Japan.

[4] Graduate School of Medical Life Science, Yokohama City University, 1-7-29 Suehiro-cho, Tsurumi, Yokohama 230-0045, Japan.

[5] Department of Mutagenesis, Division of Chromatin Regulatory Network, Radiation Biology Center, Kyoto University, Yoshidakonoe, Sakyo-ku, Kyoto 606-8501, Japan.

[6] Research Reactor Institute, Kyoto University, Kumatori, Osaka, 590-0494, Japan.

[7] RIKEN SPring-8 Center, Kouto 1-1-1, Sayo, Hyogo 679-5148, Japan.

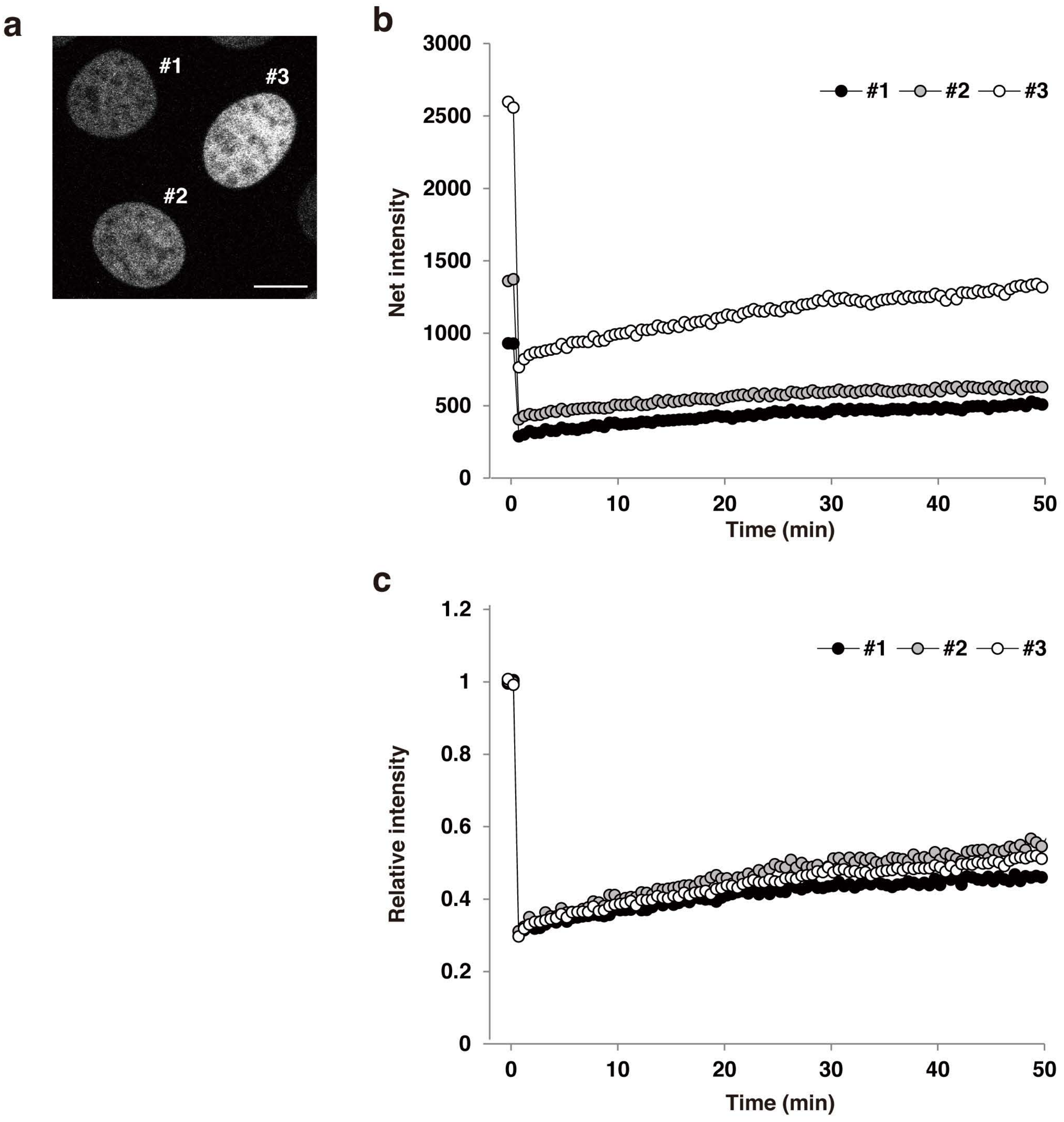

Supplementary Figure S1. FRAP recovery kinetics does not depend on the expression level of GFP-H2A.

Three nuclei with different levels of GFP-H2A expression (**a**; also shown in Fig. 2a) were analyzed as examples. The intensities of the bleached halves of nuclei and the background (outside nuclei) were measured in a time series before and after photobleaching. After subtracting the background intensity at each time point, the net intensities were plotted (**b**). Before photobleaching, cell #2 and cell #3 were ~1.5- and ~3.0-fold brighter than cell #1. To compare the FRAP recovery kinetics among different cells, the relative intensity to the initial intensity before bleaching was calculated in individual nuclei (by dividing the intensity of each time point by the average intensity before bleaching), and plotted (**c**). The FRAP recovery curves are similar for the three cells, regardless of their absolute fluorescence levels. The relative fluorescence curve of the brightest cell (cell #3) appears between those of the dimmer cells (cell #1 and cell #2), indicating that the original expression level and the recovery rate are not correlated. Bar, 10 μm.

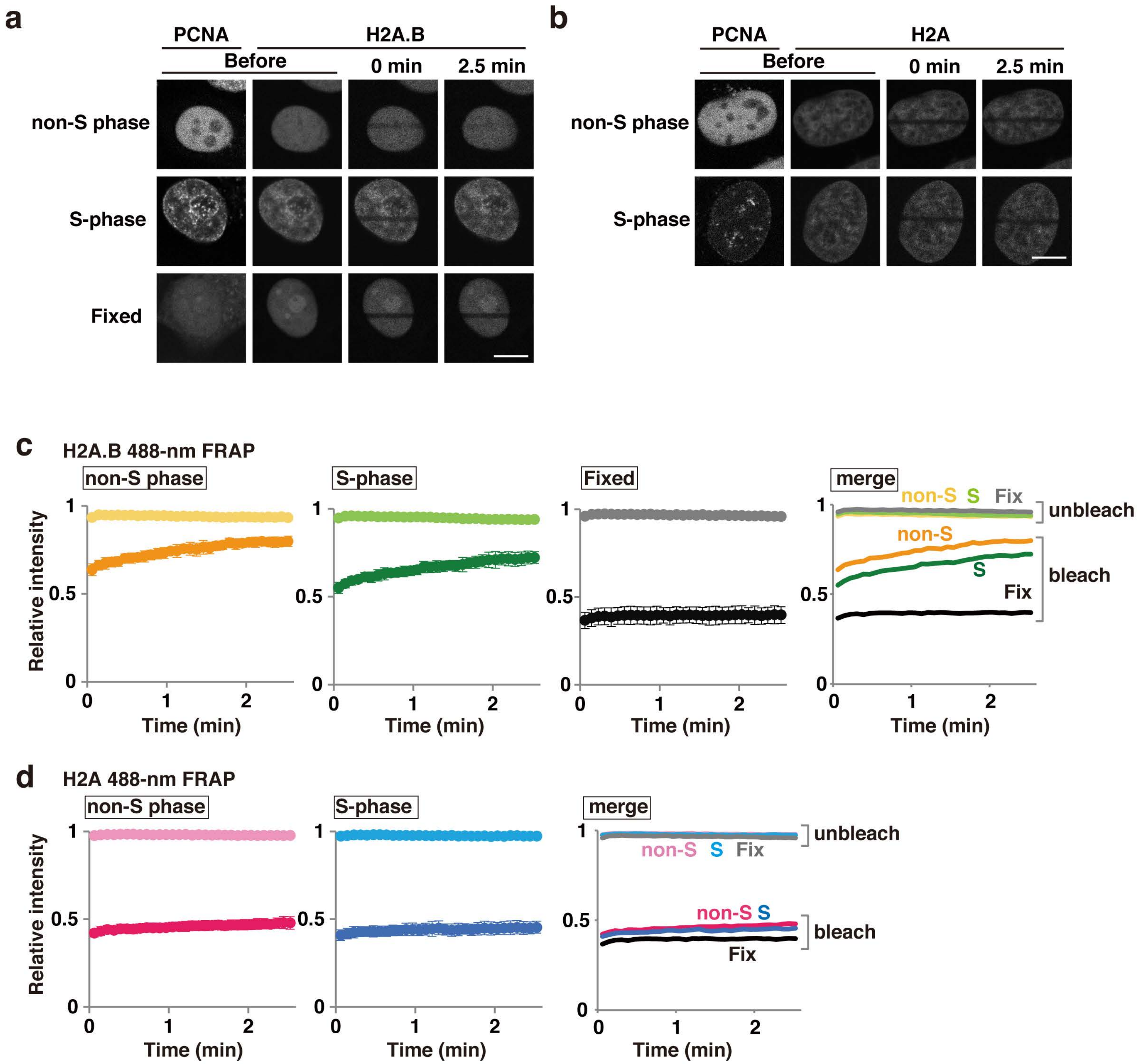

Supplementary Figure S2. The amount of the chromatin-free fraction is altered during the cell cycle, but the exchange rate of GFP-H2A.B remains unaffected.

Kinetic analyses of GFP-histones in non-S (G1 or G2) and S-phase cells were performed using HeLa cells expressing PCNA-mCherry and either GFP-H2A.B (**a**) or GFP-H2A (**b**), by strip bleaching with a 488-nm laser (110 ms). HeLa cells expressing PCNA-mCherry and GFP-H2A.B that were fixed with paraformaldehyde for 1 h were used as a control, to measure the bleach depth. To avoid inducing DNA damage, a relatively weak bleaching condition was used and ~60% was bleached in fixed cells. The cell cycle stage of individual cells was judged by the PCNA-mCherry distribution (**a** and **b**). Fluorescence intensities of bleached and unbleached areas were measured and normalized to the initial intensities in the same areas before bleaching, to obtain relative intensities. Averages with standard deviations are plotted (N = 10-14) (**c** and **d**). (**c**) GFP-H2A.B. (**d**) GFP-H2A. Graphs are also superimposed without standard deviations. Bars, 10 μm.

The recovery curves of GFP-H2A.B in the bleached areas in non-S and S-phase cells have similar shapes, but the post-bleach intensity of the bleached area in non-S phase cells is higher than that in S-phase cells. The data suggest that a larger chromatin-free, diffusible fraction is present in non-S cells. In contrast, the post-bleach intensities of GFP-H2A in both non-S and S-phase cells were just above that of the fixed cells, indicating that only a small diffusible fraction of GFP-H2A is present.

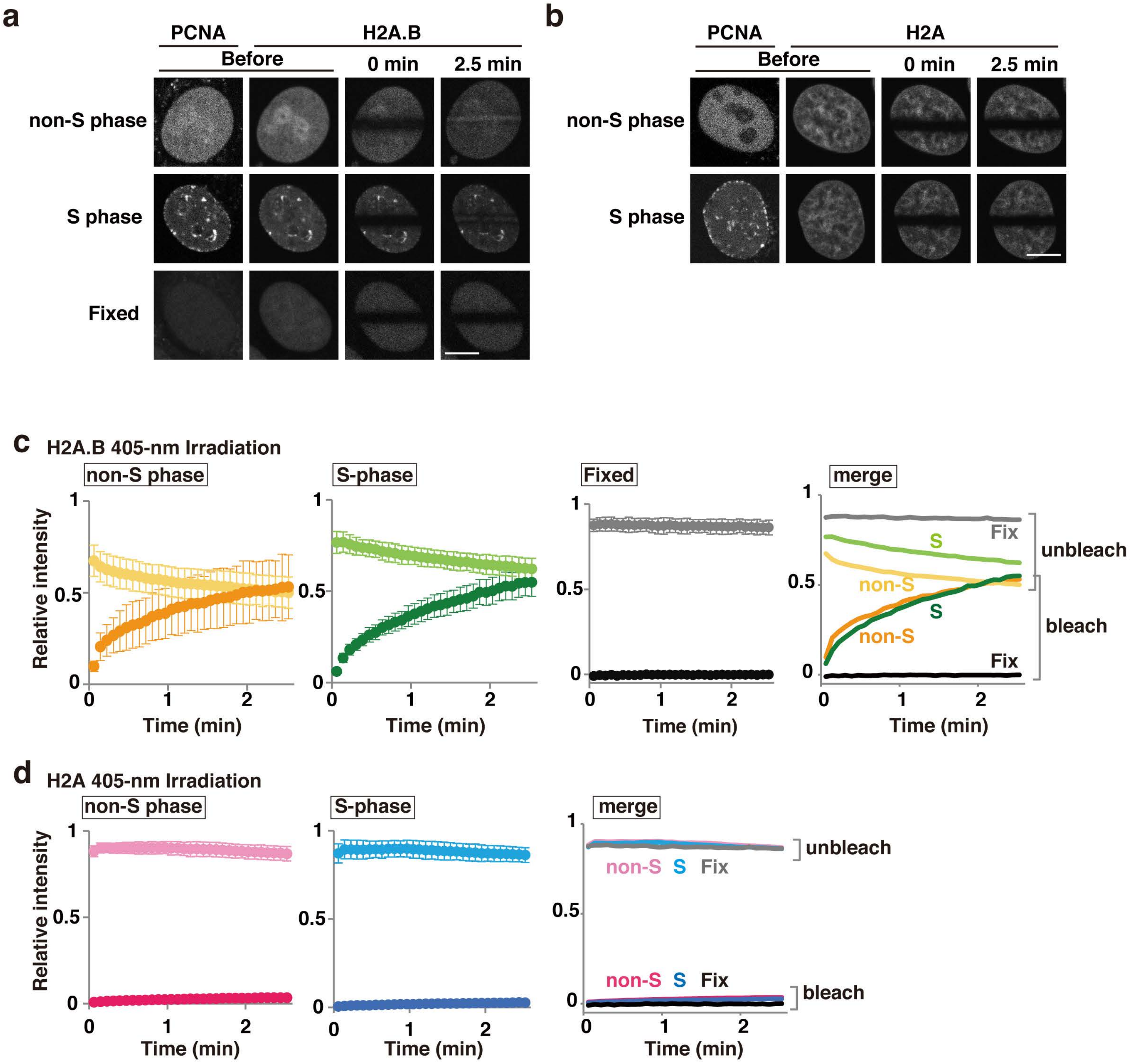

Supplementary Figure S3. GFP-H2A.B accumulates at damaged sites at similar rates in non-S and S-phase cells.

The responses of GFP-histones in non-S (G1 or G2) and S-phase cells to DNA damage were analyzed using HeLa cells expressing PCNA-mCherry and either GFP-H2A.B (**a**) or GFP-H2A (**b**), by laser irradiation with a 405-nm laser (3.63 s), which also bleaches GFP fluorescence. HeLa cells expressing PCNA-mCherry and GFP-H2A.B that were fixed with paraformaldehyde for 1 h were used as a control, to measure the bleach depth. To induce DNA damage, a strong laser irradiation was used and GFP fluorescence was completely bleached in fixed cells. The cell cycle stage of individual cells was judged by the PCNA-mCherry distribution (**a** and **b**). Fluorescence intensities of damaged and undamaged areas were measured and normalized against the initial intensities in the same areas before laser irradiation, to obtain relative intensities. Averages with standard deviations are plotted (N = 10-14) (**c** and **d**). (**c**) GFP-H2A.B. (**d**) GFP-H2A. Graphs are also superimposed without standard deviations. Bars, 10 μm.

The curves of GFP-H2A.B in the damaged areas in non-S and S-phase cells are similar, but the post-damage intensity of the undamaged area in non-S cells is lower than that in S-phase cells. Since the laser irradiation period is relatively longer than that of the bleach experiments in Supplementary Figure 2, chromatin-free molecules are bleached and the post-damage intensity of the undamaged area is also decreased. These data suggest that a larger diffusible fraction of GFP-H2A.B is present in non-S cells than in S-phase cells, consistent with the 488-nm laser bleaching. GFP-H2A in both non-S and S-phase cells does not appear to be affected by laser-induced DNA damage.

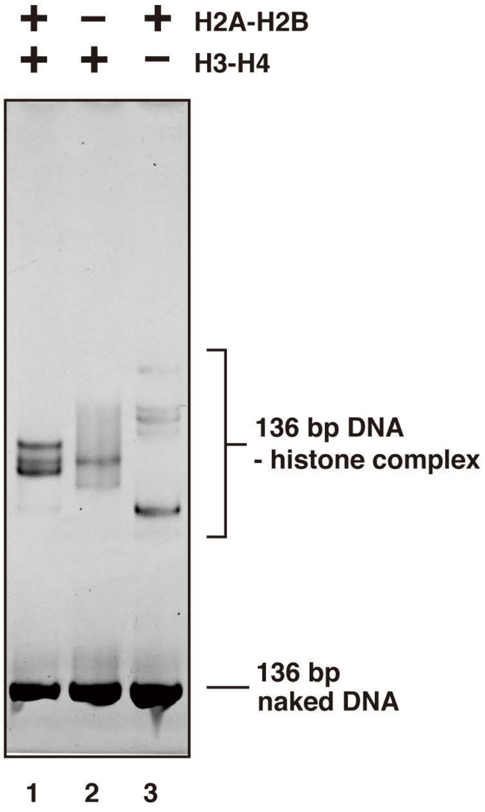

Supplementary Figure S4. Nucleosome formation assay with the histone octamer, the H3-H4 complex, or the H2A-H2B complex.

Nucleosomes reconstituted with a 136 bp DNA were analyzed by non-denaturing 6% PAGE, and the gel was stained with ethidium bromide after electrophoresis. Lanes 1-3 indicate the nucleosomes or the histone-DNA complexes with the histone octamer, the H3-H4 complex, and the H2A-H2B complex, respectively.
